# Colon cancer survival differs from right side to left side and lymph node harvest number matter

**DOI:** 10.1186/s12889-021-10746-4

**Published:** 2021-05-12

**Authors:** Lucia Mangone, Carmine Pinto, Pamela Mancuso, Marta Ottone, Isabella Bisceglia, Giorgio Chiaranda, Maria Michiara, Massimo Vicentini, Giuliano Carrozzi, Stefano Ferretti, Fabio Falcini, Cesare Hassan, Paolo Giorgi Rossi

**Affiliations:** 1 Epidemiology Unit, Azienda Unità Sanitaria Locale-IRCCS di Reggio Emilia, Via Amendola 2, 42122 Reggio Emilia, MD Italy; 2 Medical Oncology, AUSL-IRCCS di Reggio Emilia, Reggio Emilia, MD Italy; 3 Public Health Department, AUSL Piacenza, Piacenza, MD Italy; 4 Medical Oncology Unit, University Hospital of Parma, Parma, MD Italy; 5 Epidemiology Unit, Azienda Unità Sanitaria Locale, Via Martiniana 21, Baggiovara, 41126 Modena, MD Italy; 6 Romagna Cancer Registry - Section of Ferrara. Local Health Unit, University of Ferrara, Ferrara, MD Italy; 7 Romagna Cancer Registry, Istituto Scientifico Romagnolo per lo Studio e la Cura dei Tumori (IRST), IRCCS, Meldola (Forlì), Italy-Azienda Usl della Romagna, Forlì, MD Italy; 8 Endoscopy Unit, Nuovo Regina Margherita Hospital, Rome, MD Italy

**Keywords:** Colorectal cancer, Tumor side, Lymph nodes, Survival

## Abstract

**Background:**

Right-sided colorectal cancer (CRC) has worse survival than does left-sided CRC. The objective of this study was to further assess the impact of right-side location on survival and the role of the extent of lymphadenectomy.

**Methods:**

All CRCs diagnosed between 2000 and 2012 in Emilia-Romagna Region, Italy, were included. Data for stage, grade, histology, screening history, and number of removed lymph nodes (LN) were collected. Multivariable Cox regression models were used to estimate hazard ratios (HR), with relative 95% confidence intervals (95%CI), of right vs. left colon and of removing < 12, 12–21 or > 21 lymph nodes by cancer site.

**Results:**

During the study period, 29,358 patients were registered (8828 right colon, 18,852 left colon, 1678 transverse). Patients with right cancer were more often older, females, with advanced stage and high grade, and higher number of removed LNs. Five-year survival was lower in the right than in the left colon (55.2% vs 59.7%). In multivariable analysis, right colon showed a lower survival when adjusting for age, sex, and screening status (HR 1.12, 95%CI 1.04–1.21). Stratification by number of lymph nodes removed (12–21 or > 21) was associated with better survival in right colon (HR 0.54, 95%CI 0.40–0.72 and HR 0.40, 95%CI 0.30–0.55, respectively) compared to left colon (HR 0.89, 95%CI 0.76–1.06 and HR 0.83, 95%CI 0.69–1.01, respectively).

**Conclusions:**

This study confirms that right CRC has worse survival; the association is not due to screening status. An adequate removal of lymph nodes is associated with better survival, although the direction of the association in terms of causal links is not clear.

**Supplementary Information:**

The online version contains supplementary material available at 10.1186/s12889-021-10746-4.

## Background

Colorectal cancer (CRC) is the second most common malignancy in Italy (13% of cases, after breast cancer, 14%), with 49,000 new cases per year (27,000 males and 22,000 females) [1]. Currently, the right side location is associated with several negative prognostic factors: older age, advanced stage, mucinous histology [2, 3], and molecular biological profile (MMR and RAS/BRAF status), can influence prognosis and response to treatment [4].

Several retrospective studies using data from the SEER (Surveillance Epidemiology and End Results program) [5–7], other cancer registries [8], and systematic reviews [5] have shown that a higher lymph node yield is associated with better survival, especially for right-sided lesions. This observational finding is very interesting because residual confounding is expected to go in the opposite direction; the surgical removal of a higher number of lymph nodes (LNs) could be associated with more advanced disease. However, these studies analysed a period when colorectal surgical practice had changed, with a trend towards increasing the radicality of resection and the total number of LN yield [9]. This association thus suggests a causal link, and the number of LNs has been proposed as a routine indicator for quality assurance [10, 11]. Nevertheless, the mechanism underlying this association is still unclear [12]. Confirming the association in other countries may help to understand whether the underlying link is generalizable also when the proportion of patients with fewer than eleven removed LNs is already very low.

The aim of the study, using the validated data of Italian cancer registries, was to evaluate the impact of right-side location on survival and whether the surgical removal of a higher number of LNs is associated with better survival.

## Materials and methods

### Setting of the study

Data were retrieved from the Emilia-Romagna population-based pathology registry, which routinely collects all incident cases for breast, colorectal, and cervical cancers. For the reported analyses, only the area of the Bologna Local Health authority was excluded.

### Characteristics of participants

The included catchment area in 2017 had about 3,573,000 inhabitants. Included cases represent all incident cases. We included the 29,358 cases of CRC, representing all incident cases between the years 2000 and 2012. All patients were followed for at least 5 years up to December 31, 2017. Second cancers and appendix tumours were excluded.

### Data sources

Data on the date of diagnosis and, when necessary, of death, International Classification of Disease for Oncology, 3rd edition (ICD-O-3), stage, grading, histological type, surgery, lymph nodes removed, and screening status were retrieved from the Reggio Emilia Cancer Registry. The cause of death was codified according to the International Classification of Disease, 10th edition (ICD10). An analysis of tumour location was done by dividing the colon into three subsites according to ICD-O-3 [13]: the *right colon* includes cecum (C18.0), ascending (C18.2), and hepatic flexure (C18.3); the *left colon* includes splenic flexure (C18.5), descending colon (C18.6), sigma (C18.7), rectosigmoid junction (C19.9), and rectum (C20.9); the *transverse-colon* (C18.4) was analysed separately.

### Description of variables

Cancers were classified into stages I–IV according to the TNM classification [14]. Tumour grade was included as a separate variable since colorectal staging does not take grade into account. Morphology includes 4 categories: adenocarcinoma, carcinoma not otherwise specified (NOS), mucinous forms, and other. For 9684 of the cases examined, screening status was assessed: patients were divided into either uninvited or invited (screen detected, interval cancers, and non-attendees). Treatment variables were not included for this analysis, except for surgical approach (yes/no). The number of removed LNs was divided into 3 groups: < 12, 12–21, or > 21. Patients were followed from the date of diagnosis to death (for any cause), migration out of the registry catchment area, or end of study follow up (December 31, 2017), whichever occurred first. All-cause mortality was the main endpoint.

### Statistical analysis

Descriptive analyses of patient characteristics were performed by colon cancer site. The association between qualitative clinical and demographic variables and lymph nodes removed was evaluated through Pearson’s chi-squared *P*-values. Overall survival of patients with CRC was analysed by location (right vs. left vs. transverse). The Kaplan–Meier method was used to conduct an analysis of all demographic and cancer variables to trace survival over a 5-year period.

Using survival time as the main temporal axis, we fitted Cox proportional hazards models to estimate hazard ratios with 95% confidence intervals. A multivariable Cox regression analysis of overall survival was conducted to study the impact of cancer site on overall survival, adjusting for age, sex, and screening status uninvited or invited (screen detected, interval cancers, and non-attendees).

### Additional analyses

Possible confounders were identified after defining a directed acyclic graph (DAG). Models also adjusting for possible mediators are reported in the supplementary materials, together with the explanatory DAG. Further multivariable Cox regression models for overall survival were built stratified by site (excluding those without surgery, no lymph node dissection, carcinoma not otherwise specified, stage I, stage IV, and unknown) and adjusting by age, sex, histological type, grade, nodal status (according to TNM system), and screening status to measure the effect of the number of removed LNs; a sensitivity analysis including stage I was also conducted. Potential confounders were identified after defining a DAG, reported in the supplementary material.

The underlying assumptions for the Cox proportional hazards model were evaluated by visual check of the parallelism of the log-log plot of curves; furthermore, the Kaplan–Meier observed curve and the Cox predicted survival were plotted in the same graph to check for discrepancies. Linearity of the link between age and survival in the model was tested by plotting Martingale residuals. All these analyses are reported in the supplementary materials.

In the reported analyses, as we did not perform any formal statistical test of hypothesis, we did not set a statistical significance threshold; *p*-values are reported as continuous variables to measure of the probability that the observed difference would be observed under the null hypothesis of equal survival between the groups. Sample size was determined by the number of the incident cases occurring during the study period in the pathology registry catchment area, i.e., the entire Emilia-Romagna region except the Bologna Local Health Authority. We used Stata 13.0 SE (Stata Corporation, Texas, TX) software package for all the analyses.

## Results

### Right and left cancer survival

A total of 29,358 patients were eligible and included in this study: 8828 had a cancer of the right colon, 18,852 of the left colon, and 1678 of the transverse colon. We observed 16,976 (57.8%) deaths and 352 (1.2%) lost to follow up. Median and interquartile range (IQR) of follow up time were 5.2 and 1.4–9.0 years, respectively. Patient characteristics are shown in Table 1.

**Table 1:** Patient and cancer characteristics by cancer site. Incident colon cancer cases, Emilia-Romagna, Italy, 2000–2012

	Colon cancer site			
	*Right N (%)*	*Left N (%)*	*Transverse N. (%)*	Total N. (%)
Overall	8828 (30.1)	18,852 (64.2)	1678 (5.7)	29,358 (100)
Age years (SD)	73.2 (11.1)	70.3 (11.9)	72.6 (11.9)	71.3 (11.7)
Sex				
Male	4362 (49.4)	10,924 (57.9)	879 (52.4)	16,165 (55.1)
Female	4466 (50.6)	7928 (42.1)	799 (47.6)	13,193 (44.9)
Subsite				
Cecum	2729 (30.9)			
Ascending	5086 (57.6)			
Hepatic	1013 (11.5)			
Transverse			1678 (100)	
Splenic		754 (4.0)		
Descending		2608 (13.8)		
Sigmoid		7532 (40.0)		
Junction		2607 (13.8)		
Rectum		5351 (28.5)		
Stage				
Stage I	1439 (16.3)	4622 (24.5)	278 (16.6)	6339 (21.6)
Stage II	2829 (32.0)	4426 (23.5)	537 (32.0)	7792 (26.5)
Stage III	2365 (26.8)	4435 (23.5)	410 (24.4)	7210 (24.6)
Stage IV	1645 (18.6)	3365 (17.8)	341 (20.3)	5351 (18.2)
Unknown	550 (6.2)	2004 (10.6)	112 (6.7)	2666 (9.1)
Grade				
G1	669 (7.6)	1854 (9.8)	113 (6.7)	2636 (9.0)
G2	4239 (48.0)	10,055 (53.3)	881 (52.5)	15,175 (51.7)
G3–4	2581 (29.2)	3245 (17.2)	398 (23.7)	6224 (21.2)
Unknown	1339 (15.2)	3698 (19.6)	286 (17.0)	5323 (18.1)
Histological type				
Carcinoma NOS^a^	436 (4.9)	821 (4.4)	85 (5.1)	1342 (4.6)
Adenocarcinoma	7263 (82.3)	17,002 (90.2)	1413 (84.2)	25,678 (87.5)
Mucinous	1092 (12.4)	1013 (5.4)	171 (10.2)	2276 (7.8)
Other	37 (0.4)	16 (0.2)	9 (0.5)	62 (0.2)
Surgery				
No	423 (4.8)	856 (4.5)	81 (4.8)	1360 (4.6)
Yes	8405 (95.2)	17,996 (95.5)	1597 (95.2)	27,998 (95.4)
Lymph nodes				
Lymphadenectomy ^b^	7617 (90.6)	14,198 (78.9)	1385 (82.5)	23,200 (82.9)
< 12^c^	1173 (14.0)	4783 (26.6)	402 (24.0)	6358 (22.7)
12–21 ^c^	3663 (43.6)	6344 (35.3)	591 (35.2)	10,598 (37.9)
> 21 ^c^	2781 (33.1)	3071 (17.1)	392 (23.4)	6244 (22.3)
Screening				
Uninvited	327 (12.6)	963 (14.7)	64 (12.1)	1354 (14.0)
Invited	2267 (87.4)	5598 (85.3)	465 (87.9)	8330 (86.0)
Screen detected	873 (38.5)	2529 (45.2)	209 (44.9)	3611 (43.3)
Interval cancer	461 (20.3)	639 (11.4)	74 (15.9)	1174 (14.1)
Non-attendees	933 (41.2)	2430 (43.4)	182 (39.1)	3545 (42.6)

^a^not otherwise specified

^b^percentages on patients with surgery

^c^percentages on patients with at least one lymphadenectomy

**Fig. 1:**
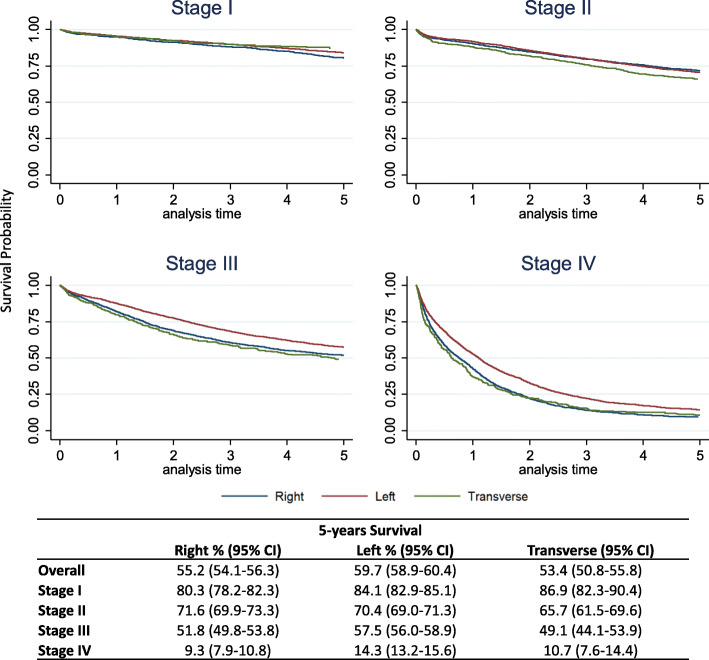
Five-year survival by stage and cancer site. Curves and percentage of survivors 5 years after diagnosis, with relative 95% confidence intervals (95%CI), were computed using Kaplan Meier estimator. Incident colon cancer cases, Emilia-Romagna, Italy, 2000–2012, follow up until December 31, 2017

Right-sided CRC was more frequent in older age, among females, at slightly more advanced stages, higher grade, and in mucinous forms than was left-sided CRC. Screen-detected tumours prevailed in the left colon (45.2%), while interval cancers prevailed in the right colon (20.3%). Five-year survival (Fig. 1) showed lower values for the right than for the left colon, overall (55.2% vs 59.7%) and for stages I (80.3% vs 84.1%), stage III (51.8% vs 57.5%), and IV (9.3% vs 14.3%). Only in stage II did the right colon have slightly higher survival values (71.6% vs 70.4%). The transverse site showed a higher survival in stage I (86.9%) but worse than the other two sites for stages II (65.7%) and III (49.1%); stage IV had intermediate values (10.7%).

After the exclusion of patients not classifiable for screening status (12,621 out of screening age, i.e., < 50 or > 70, and 7033 cases occurring before the start of the regional screening program in March 2005), multivariable analysis adjusted for age, sex, and screening status (screened vs. non-screened) showed that prognosis was worse for right colon than for left colon (HR 1.12 [95%CI 1.04–1.21]) (Table 2).

**Table 2:** Multivariable Cox proportional hazard model reporting hazard ratios for cancer site adjusted for age, sex, and screening status (*n* = 9674)

Factors	Multivariable analysis				
	*n*	*Person years*	*HR*	95% CI	*P*-value
*Cancer site*					
Left	2298	44,034.3	1		
Right	988	16,050.8	1.12	(1.04–1.21)	0.003
Transverse	182	3353.8	1.05	(0.90–1.22)	0.555
*Age*			1.04	(1.03–1.04)	< 0.001
*Sex*					
Male	2219	36,624.7	1		
Female	1249	26,814.2	0.80	(0.74–0.85)	< 0.001
*Screening status*					
Unscreened (uninvited/ non-attendees)	2292	29,003.0	1		
Screened (Screen-detected/interval cancers)	1176	34,435.9	0.45	(0.42–0.48)	< 0.001

Incident colon cancer cases, Emilia-Romagna, Italy, 2000–2012, follow up until December 31, 2017

When stage and grade (excluding grade unknown) were included in the model (supplementary Table 1), survival was higher for right colon than for left colon (HR 1.11 [95%CI 1.02–1.21]). When the number of removed LNs (excluding patients not surgically treated) was also included in the model, the excess in right colon increased (HR 1.22 [95%CI 1.12–1.33]) (supplementary Table 3).

### Lymphadenectomy and survival

The effect on prognosis of the number of surgically removed LNs was another aim of our study. This analysis was limited to the 23,269 cases of non-metastatic cancer with surgical resection. Table 3 shows that the number of LNs harvested was slightly higher in the right colon and in the transverse colon, while it was less frequent in stage I, and virtually absent for carcinoma NOS (which were therefore excluded from the multivariable analysis). The proportion of patients with fewer than 12 removed LNs was 14.3 and 27.9% in right and left colon, respectively, but for < 12 Ln and > 21 Ln the percentage values are inverted.

**Table 3:** Patient and cancer characteristics by number of lymph nodes removed. Incident non-metastatic colon cancer cases who underwent surgery, Emilia-Romagna, Italy, 2000–2012

Variable	Total	No lymphadenectomy		LN h < 12		LN h 12–21		LN h > 21		*P*-value^*c*^
	*N.*	*N. (%)*		*N. (%)*		*N. (%)*		*N. (%)*		
	23,269	3330	(14.3)	5533	(23.8)	9107	(39.1)	5299	(22.8)	
*Site*										< 0.001
Left	15,006	2759	(18.4)	4200	(27.9)	5481	(36.5)	2566	(17.1)	
Right	6962	451	(6.5)	992	(14.3)	3127	(44.9)	2392	(34.4)	
Transverse	1301	120	(9.2)	341	(26.2)	499	(38.4)	341	(26.2)	
*Sex*										< 0.001
Male	12,936	1866	(14.4)	3218	(24.9)	5121	(38.6)	2731	(21.1)	
Female	10,333	1464	(14.2)	2315	(22.4)	3986	(38.6)	2568	(24.9)	
*(Histological type)*										< 0.001
Carcinoma NOS^a^	256	204	(79.7)	22	(8.6)	20	(7.8)	10	(3.9)	
Adenocarcinoma	21,142	3053	(14.4)	5109	(24.2)	8247	(39.0)	4733	(22.4)	
Mucinous	1822	69	(3.8)	396	(21.7)	824	(45.2)	533	(29.3)	
Other	49	4	(8.2)	6	(12.2)	16	(32.7)	23	(46.9)	
*Screening status*										< 0.001
Uninvited	1073	108	(10.1)	237	(22.1)	417	(38.9)	311	(29.0)	
Screen-detected + IC^b^	4263	631	(14.8)	1059	(24.8)	1667	(39.1)	906	(21.2)	
Non-attendees	2552	282	(11.1)	453	(17.7)	1022	(40.1)	795	(31.2)	
*Stage*										< 0.001
Stage I	6315	1358	(21.5)	2042	(32.3)	2051	(32.5)	864	(13.7)	
Stage II	7755	65	(0.8)	1721	(22.2)	3622	(46.7)	2347	(30.3)	
Stage III	7174	57	(0.8)	1680	(23.4)	3370	(47.0)	2067	(28.8)	
Unknown	2025	1850	(91.4)	90	(4.4)	64	(3.2)	21	(1.04)	
*Grade*										< 0.001
G1	2492	657	(26.4)	676	(27.1)	747	(30.0)	412	(16.5)	
G2	13,142	877	(6.7)	3327	(25.3)	5721	(43.5)	3217	(24.5)	
G3–4	4657	256	(5.5)	967	(20.8)	2048	(44.0)	1386	(29.8)	
Unknown	2978	1540	(51.7)	563	(18.9)	591	(19.9)	284	(9.5)	

^a^Carcinoma not otherwise specified

^b^ Interval cancers

^c^Pearson’s chi-squared test

The multivariable analysis, stratified by site (Table 4), showed that the removal of 12–21 LNs from the right colon was associated with better survival (HR 0.54 [95%CI 0.40–0.72]) and that the HR further decreased when more than 21 LNs were removed (HR 0.40 [95%CI 0.30–0.55]). This was true for the left colon as well, although the association was weaker for 12–21 LNs harvested (HR 0.89 [95%CI, 0.76–1.06]) and for more than 21 LNs (HR 0.83 [95%CI 0.69–1.01]).

**Table 4:** Multivariable Cox proportional hazard models reporting hazard ratios for number of removed lymph nodes by cancer site

*Factors*	Right					Left				
	n	Person years	HR	(95%CI)	*P-*value	n	Person years	HR	(95%CI)	*P-*value
LN harvest										
< 12	58	664.5	1			210	4528.7	1		
12–21	201	4294.4	0.54	(0.40–0.72)	< 0.001	396	9514.8	0.89	(0.76–1.06)	0.194
> 21	169	4906.2	0.40	(0.30–0.55)	< 0.001	218	5737.9	0.83	(0.69–1.01)	0.064
Stage										
II	167	5547.2	1			327	9970.5	1		
III	261	4317.9	2.01	(1.65–2.44)	< 0.001	497	9810.8	1.59	(1.38–1.83)	< 0.001

The models are adjusted for sex, age, histotype, screening status, stage, and grading

Incident colon cancer cases with lymphadenectomy, stages II and III, Emilia-Romagna, Italy, 2000–2012, follow up until December 31, 2017

A sensitivity analysis was also conducted that included stage I cases (data not shown): the HR for LN harvest in the right colon was almost identical to that when stage I was excluded, while for the left colon the HR was 0.97 [95%CI 0.84–1.12] for a yield of 12–21 LNs and 0.88 [95%CI 0.74–1.05] for a yield of > 21 LNs.

## Discussion

Our results confirm that right-sided CRC is associated with worse five-year survival overall than is left-sided CRC. Differences in survival were appreciable for stages I, III, and IV, but not for stage II. While the overall worse survival of right colon cancer has been reported by almost all studies, stage-specific results are inconsistent: Lee and colleagues found a difference only for stages II and III [5], Lim and colleagues only for stage III [15], and Loupakis and colleagues only for metastatic cancers [16]. Weiss and colleagues also confirmed worse prognosis in the right colon (only for stage III), although their study included only patients over age 65 [17].

The patients in our study with right-sided CRC were more often elderly, female, with advanced stage, high grade, and a greater number of lymph nodes removed. These values are in line with those reported in the literature [5, 15, 18]. Surprisingly, in our population-based case series, multivariable analyses did not show worse survival for right colon cancer by age and sex. The survival of right-sided CRC resulted lower than that of left-sided CRC only when also adjusting for screening history (HR1.12 [95%CI 1.04–1.21]. It is worth noting that screening changes the distribution of right- and left-sided cancers through the identification and treatment of cancer precursors, which leads to the prevention especially of left-sided cancers [19], but screening is also associated with better survival through early diagnosis and stage shift, decreasing the hazard of participants by about 50%. We did not included stage, grade, and extent of lymphadenectomy in our main model since they could be mediators of the effect of screening and cancer site. Nevertheless, it is interesting to note that the difference in survival between right and left colon became stronger (HR 1.22 [95%CI 1.12-1.33]) when we adjusted for the number of lymph nodes removed, stage, and grade, principally because, on average, more lymph nodes are removed for right-sided cancers and because more lymph nodes are associated with better survival. These values are in line with those reported by Yahagi [20] (pooled HR 1.14 [95%CI 1.06–1.22]) and confirmed by Lee (HR 1.12 [95%CI 1.06–1.19]) [5] in population-based studies and by Petrelli and colleagues (HR 0.82 for left vs right colon, [95%CI 0.79–0.84]) in a meta-analysis that included about 14 million patients from both trials and retrospective cohort studies [4].

Our data suggest that differences in survival between right and left colon cancer cannot be due to early diagnosis or to the surgical procedure used because when we adjusted for these factors, the difference in survival between left and right colon cancer increased. Excluding any role of early diagnosis as well as any role of the difference between surgical procedures, our analyses support the hypothesis of a major role for other possible causes, such as the different distribution of the molecular profiles [18, 19] and/or different microbiota [21–23].

We confirmed a strong association between lymph node removal and survival. The multivariable analysis showed that in the right colon, removal of 12–21 LNs was associated with better survival (HR 0.53 [95%CI 0.40–0.72]) and that the HR was further reduced when more than 21 LNs were removed (HR 0.40 [95%CI 0.30–0.55]).

These values are even stronger than those reported by Lee and colleagues, who found HRs for right-sided CRC of 0.71 and of 0.61 with the removal of 12–21 and of more than 22 LNs, respectively [5].

Xie and colleagues also add that the number of LNs examined and the number of positive LNs are an important prognostic factor: right-sided CRC with an increased number of LNs examined and adequate LN harvest at diagnosis is associated with decreased risk of LN positivity [24]. Ng and colleagues proposed a formula to quantify the number of LNs to be removed, which varies according to age, location, and size [25]. A systematic review of 17 studies from 1990 to 2006 that included 61,371 patients reported a positive association between a higher nodal harvest and long-term outcomes [26].

It is unclear whether this association was due to stage migration, or whether the number of removed LNs is a biomarker of some biological characteristic affecting prognosis, or whether we are observing the therapeutic impact of lymph node removal itself [12]. The emerging evidence in favour of complete mesocolic excision [27], which includes a higher LN yield than other surgical techniques, makes the hypothesis of a therapeutic effect plausible. However, it is unlikely that complete mesocolic excision had a major role in this study because before 2012, its use was not common. It is worth noting that in our population we observed the lowest proportion of patients with fewer than 12 removed LNs, i.e., 14%, compared to other studies [5, 26], and at the same time the strongest association with an HR for more than 22 LNs vs fewer than 12 LNs. Indeed, an inverse relation between the prevalence of the group with worse prognosis and the strength of the association suggests that we are dealing with a biomarker, i.e., a low number of LNs dissected, characterizing a small group of patients with very poor prognosis, and that the spread of surgical techniques involving extended lymphadenectomy are making the biomarker more and more specific. Nevertheless, although the causal pathway remains unclear [28], our data call into question the current 12 lymph node minimum standard and may provide indirect evidence of complete mesocolic excision to obtain a higher lymphatic harvest to improve survival.

### Strengths and limitations

Our study does not include data on the type of surgery, postoperative complications, the specific chemotherapy regimen used, or systemic therapy compliance, which may affect oncologic outcomes [29]. Nevertheless, less than 5% of our cases overall did not undergo surgery, with no distinction between right-sided, left-sided, or transverse CRC.

There is broad variability in the definition of *right* and *left colon* (supplementary Fig. 10) [2–5, 15–18, 24, 25, 30]; we decided to follow the definition used by 5 recent studies [2, 4, 16, 18, 25] (including rectum and junction), considering the transverse colon separately. Nevertheless, comparability between studies is limited by the heterogeneity of definitions.

Ours was a population-based study using cancer registry data for an entire region in Italy rather than from a single centre. We were also able to adjust for screening status in a region where a CRC screening programme has been active since 2005, with a 50% participation rate [31]. Screening has changed the epidemiological profile of colorectal cancer in Italy and has also had an impact on survival [2, 20]. Furthermore, our study population had a very low proportion of patients with fewer than 12 LNs removed. These two characteristics make our results generalizable to the epidemiological and clinical situation that most European countries are now facing.

## Conclusions

Right-sided colon cancer is associated with outcomes that are worse than those of left-sided CRC only when adjusting for the screening history. Furthermore, the worse survival is not mediated by the number of LNs removed. Thus, our analyses suggest that worse survival is not due to early diagnosis or to differences in surgical procedures. We also confirm that a higher lymph node yield is associated with better survival, especially for right-sided lesions, even in a population where more than 12 LNs are removed for the vast majority of right-sided CRC. Our results, read in the context of the current literature, suggest that any association between LN harvest and survival should be interpreted as a non-causal association, i.e., LN harvesting is a biomarker, rather than as a causal therapeutic effect. Nevertheless, we cannot exclude either of the two hypotheses.

## Supplementary Information

**Additional file 1: Supplementary Table 1.** Person years, number of deaths and censored (moved out of the cancer registry area) by cancer site, patient, and cancer characteristics. Incident colon cancer cases, Emilia-Romagna, Italy, 2000-2012, follow up to December 31, 2017. **Supplementary Figure 1.** Directed acyclic graph of the putative causal pathway linking colon cancer site with cancer survival. **Supplementary Figure 2.** Directed acyclic graph of the putative causal pathway linking number of removed lymph nodes in colon cancer surgery with cancer survival. **Supplementary Figure 3.** Log-log plot of survival by colon cancer side. Curves are constantly parallel after the first month of follow up, i.e., ln -2.5years of follow up. **Supplementary Figure 4.** Comparison of the observed Kaplan–Meier survival curves and Cox proportional hazard predicted survival curves. For all three groups (right, left, and transvers), observed and predicted curves substantially overlap. **Supplementary Figure 5.** Plots of Martingale residuals computed for age. The plot of Martingale residuals is random, showing no systematic patterns or trends, and the LOESS smoothed curve appears to lie around a horizontal line through zero, supporting the linear component of the age variable correctly describing the effect of age on survival. **Supplementary Figure 6.** Log-log plot of survival curves by number of lymph nodes, right-sided cancers. Curves are constantly parallel throughout follow up. **Supplementary Figure 7.** Comparison of the observed Kaplan–Meier survival curves and Cox proportional hazard predicted survival curves, right-sided cancers. For all the three groups (<12, 12-21, >21 removed lymph nodes), observed and predicted curves substantially overlap. **Supplementary Figure 8.** Log-log plot of survival curves by number of lymph nodes, left-sided cancers. Curves are constantly parallel throughout follow up. **Supplementary Figure 9.** Comparison of the observed Kaplan–Meier survival curves and Cox proportional hazard predicted survival curves, left-sided cancers. For all the three groups (<12, 12-21, >21 removed lymph nodes), observed and predicted curves substantially overlap. **Supplementary Table 2.** Multivariable Cox proportional hazard model reporting hazard ratios for cancer site adjusted for age, sex, screening status, stage, and grade. Incident colon cancer cases, Emilia-Romagna, Italy, 2000-2012, follow up until December 31, 2017. Number of observations=8,327. **Supplementary Table 3.** Multivariable Cox proportional hazard model reporting hazard ratios for cancer site adjusted for age, sex, screening status, stage, grade, and number of removed lymph nodes. Incident colon cancer cases, Emilia-Romagna, Italy, 2000-2012, follow up until December 31, 2017. Number of observations=8,274. **Supplementary Figure 10.** Definition of left and right colon as reported in recently published studies.

## Data Availability

The data are available at the Cancer Registry of Romagna, Responsible for Dr. Fabio Falcini. The data can be requested to the Istituto Oncologico Romagnolo sending in the person of Dr. Falcini (fabio.falcini@irst.emr.it) and upon authorization of the Regional Screening Coordinating Group; only aggregated data can be given; individual data can be analysed by third institutions only after the approval of a research protocol by the three competent ethics committees: Comitato Etico Area Vasta Emilia Nord (CEReggioEmilia@ausl.re.it), Comitato Etico Area Vasta Emilia Centro (ricerca@ospfe.it), and Comitato Etico Romagna (segreteriamministrativa.ceav@irst.emr.it).
