# Supplementary material for: Colon cancer survival differs from right side to left side and lymph node harvest number matter
Source: BMC Public Health. 2021 May 12;21:906. doi: 10.1186/s12889-021-10746-4 (PMC8117551; doi:10.1186/s12889-021-10746-4)
Supplement: Supplementary file 1 — Additional file 1: Supplementary Table 1. Person years, number of deaths and censored (moved out of the cancer registry area) by cancer site, patient, and cancer characteristics. Incident colon cancer cases, Emilia-Romagna, Italy, 2000-2012, follow up to December 31, 2017. Supplementary Figure 1. Directed acyclic graph of the putative causal pathway linking colon cancer site with cancer survival. Supplementary Figure 2. Directed acyclic graph of the putative causal pathway linking number of removed lymph nodes in colon cancer surgery with cancer survival. Supplementary Figure 3. Log-log plot of survival by colon cancer side. Curves are constantly parallel after the first month of follow up, i.e., ln -2.5years of follow up. Supplementary Figure 4. Comparison of the observed Kaplan–Meier survival curves and Cox proportional hazard predicted survival curves. For all three groups (right, left, and transvers), observed and predicted curves substantially overlap. Supplementary Figure 5. Plots of Martingale residuals computed for age. The plot of Martingale residuals is random, showing no systematic patterns or trends, and the LOESS smoothed curve appears to lie around a horizontal line through zero, supporting the linear component of the age variable correctly describing the effect of age on survival. Supplementary Figure 6. Log-log plot of survival curves by number of lymph nodes, right-sided cancers. Curves are constantly parallel throughout follow up. Supplementary Figure 7. Comparison of the observed Kaplan–Meier survival curves and Cox proportional hazard predicted survival curves, right-sided cancers. For all the three groups (<12, 12-21, >21 removed lymph nodes), observed and predicted curves substantially overlap. Supplementary Figure 8. Log-log plot of survival curves by number of lymph nodes, left-sided cancers. Curves are constantly parallel throughout follow up. Supplementary Figure 9. Comparison of the observed Kaplan–Meier survival curves and Cox proportiona [file 12889_2021_10746_MOESM1_ESM.docx]

**Colon cancer survival differs from right side to left side and lymph node harvest number matter**

Lucia Mangone^1^, MD, Carmine Pinto^2^, MD, Pamela Mancuso^1^, MSc, Marta Ottone^1^, MSc, Isabella Bisceglia^1^, MSc, Giorgio Chiaranda^3^, MD, Maria Michiara^4^, MD, Massimo Vicentini^1^, MSc, Giuliano Carrozzi^5^, MD, Stefano Ferretti^6^, MD, Fabio Falcini^7^, MD, Cesare Hassan^8^, MD, Paolo Giorgi Rossi ^1^, PhD

^1^ Epidemiology Unit, Azienda Unità Sanitaria Locale-IRCCS di Reggio Emilia, Via Amendola 2, 42122 Reggio Emilia, Italy, MD

^2^ Medical Oncology, AUSL-IRCCS di Reggio Emilia, Reggio Emilia, Italy, MD

^3^ Public Health Department, AUSL Piacenza, Italy, MD

^4^ Medical Oncology Unit, University Hospital of Parma, Parma, Italy, MD

^5^ Epidemiology Unit, Azienda Unità Sanitaria Locale, Via Martiniana 21, 41126 Baggiovara (Modena), Italy, MD

^6^ Romagna Cancer Registry - Section of Ferrara. Local Health Unit, University of Ferrara, Ferrara, Italy, MD

^7^ Romagna Cancer Registry, Istituto Scientifico Romagnolo per lo Studio e la Cura dei Tumori (IRST), IRCCS, Meldola (Forlì), Italy-Azienda Usl della Romagna, Forlì, Italy, MD

^8^ Endoscopy Unit, Nuovo Regina Margherita Hospital. Rome, Italy, MD

*Corresponding author*

Lucia Mangone

Epidemiology Unit, AUSL - IRCCS di Reggio Emilia,

Via Amendola 2, 42122, Reggio Emilia, Italy

^e-mail:^ [^mangone.lucia@ausl.re.it^](mailto:mangone.lucia@ausl.re.it)

Supplementary Table 1. Person years, number of deaths and censored (moved out of the cancer registry area) by cancer site, patient, and cancer characteristics. Incident colon cancer cases, Emilia-Romagna, Italy, 2000-2012, follow up to December 31, 2017.

|  |  | **Deaths** | | | | **Censored** | | | |
| --- | --- | --- | --- | --- | --- | --- | --- | --- | --- |
|  | Person years | Total N. (%) | *Right N (%)* | *Left N (%)* | *Transverse (N. (%)* | Total N. (%) | *Right N (%)* | *Left N (%)* | *Transverse N. (%)* |
| Overall | 161,450 | 16,885 (100%) | 5,264 (31.2%) | 10,615 (62.9%) | 1,006 (6.0%) | 352 (100%) | 101 (28.7) | 226 (64.2) | 25 (7.1) |
| Age years (SD) |  | 75.1 (11.0) | 76.4 (10.3) | 74.3 (11.2) | 76.4 (11.0) | 67.4 (14.3) | 71.1 (12.7) | 66.1 (14.9) | 64.5 (12.9) |
| Sex |  |  |  |  |  |  |  |  |  |
| Male | 88,784 | 9,560 (56.6%) | 2,684 (51.0%) | 6,341 (59.7%) | 535 (53.2%) | 179 (50.9) | 42 (41.6) | 121 (53.5) | 16 (64.0) |
| Female | 72,666 | 7,325 (43.4%) | 2,580 (49.0%) | 4,274 (40.3%) | 471 (46.8%) | 173 (49.2) | 59 (58.4) | 105 (46.5) | 9 (36.0) |
| Stage |  |  |  |  |  |  |  |  |  |
| Stage I | 49,749 | 2,060 (12.2%) | 504 (9.6%) | 1,483 (14.0%) | 73 (7.3%) | 82 (23.3) | 19 (18.8) | 61 (27.0) | 2 (8.0) |
| Stage II | 52,394 | 3,776 (22.4) | 1,344 (25.5%) | 2,157 (20.3%) | 275 (27.3%) | 118 (33.5) | 44 (43.6) | 61 (27.0) | 13 (52.0) |
| Stage III | 40,458 | 4,184 (24.8%) | 1,435 (27.3%) | 2,497 (23.5%) | 252 (25.1%) | 94 (26.7) | 23 (22.8) | 62 (27.4) | 9 (36.0) |
| Stage IV | 1,0371 | 4,898 (29.0%) | 1,537 (29.2%) | 3,047 (28.7%) | 314 (31.2%) | 24 (6.8) | 9 (8.9) | 14 (6.2) | 1 (4.0) |
| Unknown | 8,479 | 1,967 (11.7%) | 444 (8.4%) | 1,431 (13.5%) | 92 (9.2%) | 34 (9.7) | 6 (5.9) | 28 (12.4) | 0 (0) |
| Grade |  |  |  |  |  |  |  |  |  |
| G1 | 19,081 | 1,075 (6.4%) | 299 (5.7%) | 732 (6.9%) | 44 (4.4%) | 44 (12.5) | 7 (6.9) | 36 (15.9) | 1 (4.0) |
| G2 | 94,187 | 7,867 (46.6%) | 2,234 (42.4%) | 5,151 (48.5%) | 482 (47.9%) | 176 (50.0) | 53 (52.5) | 108 (47.8) | 15 (60.0) |
| G3-4 | 29,538 | 4,045 (24.0%) | 1,694 (32.2%) | 2,098 (19.8%) | 253 (25.2%) | 68 (19.3) | 24 (23.8) | 35 (15.5) | 9 (36.0) |
| Unknown | 18,643 | 3,898 (23.1%) | 1,037 (19.7%) | 2,634 (24.8%) | 227 (22.6%) | 64 (18.2) | 17 (16.8) | 47 (20.8) | 0 (0) |
| Histological type |  |  |  |  |  |  |  |  |  |
| Carcinoma NOS^a^ | 1,793 | 1,173 (7.0%) | 379 (7.2%) | 715 (6.7%) | 79 (7.9%) | 18 (5.1) | 3 (3.0) | 15 (6.6) | 0 (0) |
| Adenocarcinoma | 147,554 | 14,224 (84.2%) | 4,201 (79.8%) | 9,212 (86.8%) | 811 (80.6%) | 305 (86.7) | 82 (81.2) | 202 (89.4) | 21 (84.0) |
| Mucinous | 11,805 | 1,457 (8.6%) | 668 (12.7%) | 677 (6.4%) | 112 (11.1%) | 27 (7.7) | 15 (14.9) | 8 (3.5) | 4 (16.0) |
| Other | 298 | 31 (0.2%) | 16 (0.3%) | 11 (0.1%) | 4 (0.4%) | 2 (0.6) | 1 (1.0) | 1 (0.4) | 0 (0) |
| Surgery |  |  |  |  |  |  |  |  |  |
| No | 1,666 | 1,194 (7.1%) | 389 (7.4%) | 730 (6.9%) | 75 (7.5%) | 21 (6.0) | 3 (3.0) | 18 (8.0) | 0 (0) |
| Yes | 159,784 | 15,691 (92.9%) | 4,875 (92.6%) | 9,885 (93.1%) | 931 (92.5%) | 331 (94.0) | 98 (97.0) | 208 (92.0) | 25 (100) |
| Lymph nodes |  |  |  |  |  |  |  |  |  |
| Removed | 141,187 | 12,409 (73.5%) | 4,248 (80.7%) | 7,395 (69.7%) | 766 (76.1%) | 285 (81.0) | 87 (86.1) | 173 (76.5) | 25 (100) |
| < 12 | 38,269 | 3,633 (29.3%) | 794 (18.7%) | 2,585 (35.0%) | 254 (33.2%) | 47 (16.5) | 10 (11.5) | 34 (19.7) | 3 (12.0) |
| 12-21 | 63,571 | 5,774 (46.5%) | 2,120 (49.9%) | 3,330 (45.0%) | 324 (42.3%) | 136 (47.7) | 41 (47.1) | 84 (48.6) | 11 (44.0) |
| > 21 | 39,348 | 3,002 (24.2%) | 1,334 (31.4%) | 1,480 (20.0%) | 188 (24.5%) | 102 (35.8) | 36 (41.4) | 55 (31.8) | 11 (44.0) |
| Screening |  |  |  |  |  |  |  |  |  |
| Not invited | 10,280 | 605 (17.5%) | 141 (14.3%) | 434 (18.9%) | 30 (16.5%) | 25 (21.9) | 8 (25.8) | 17 (21.8) | 0 |
| Invited | 53,159 | 2,863 (82.6%) | 847 (85.7%) | 1,864 (81.1%) | 152 (83.5%) | 89 (78.1) | 23 (74.2) | 61 (78.2) | 5 (100) |
| Screen-detected | 28,125 | 744 (26.0%) | 205 (24.2%) | 499 (26.8%) | 40 (26.3%) | 31 (34.8) | 7 (30.4) | 24 (39.3) | 0 |
| Interval cancer | 6,311 | 432 (15.1%) | 183 (21.6%) | 222 (11.9%) | 27 (17.8%) | 3 (3.4) | 2 (8.7) | 1 (1.6) | 0 |
| Non-attendees | 18,723 | 1,687 (58.9%) | 459 (54.2%) | 1,143 (61.3%) | 85 (55.9%) | 55 (61.8) | 14 (60.9) | 36 (59.0) | 5 (100) |

^a^not otherwise specified; SD: standard deviation

**Supplementary** **Figure 1. Directed acyclic graph of the putative causal pathway linking colon cancer site with cancer survival.**


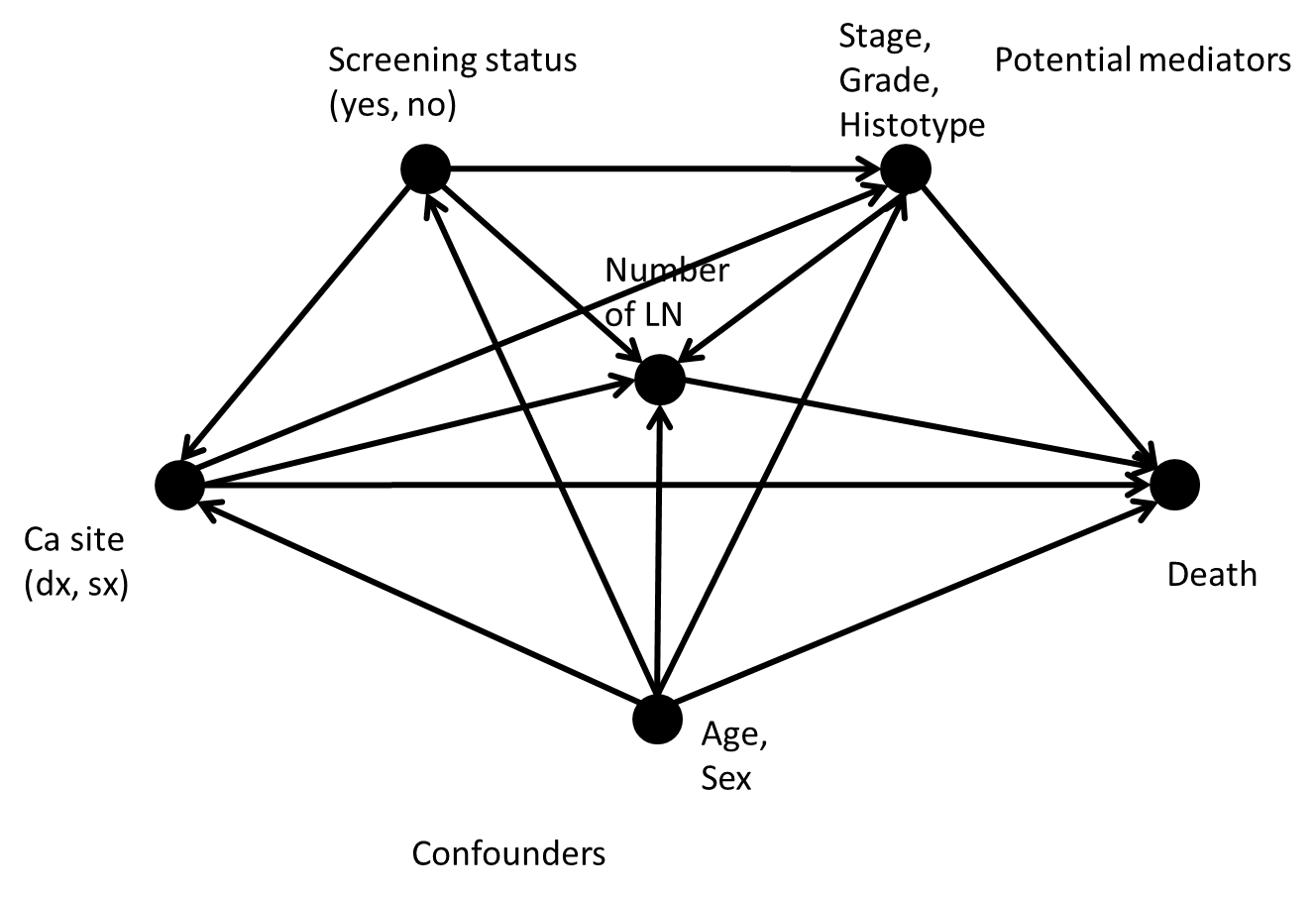


**Legend**.

Cancer site is influenced by screening (screening reduces the incidence of left cancers), age, and sex (right cancers are more common in older subjects and in females); cancer site influences the probability of early detection (left side usually detected at earlier stage), and is also associated with the number of removed lymph nodes; age and sex are possible confounders since they are associated also with death, screening status, stage and grade, and the number of removed lymph nodes; stage, grade, and histotype are possible mediators of the effect of screening on survival, and the number of removed lymph nodes may be a mediator of the effect of screening and of the cancer site. Finally, the number of lymph nodes removed may also mediate the effect of stage on mortality.

**Supplementary figure 2. Directed acyclic graph of the putative causal pathway linking number of removed lymph nodes in colon cancer surgery with cancer survival.**


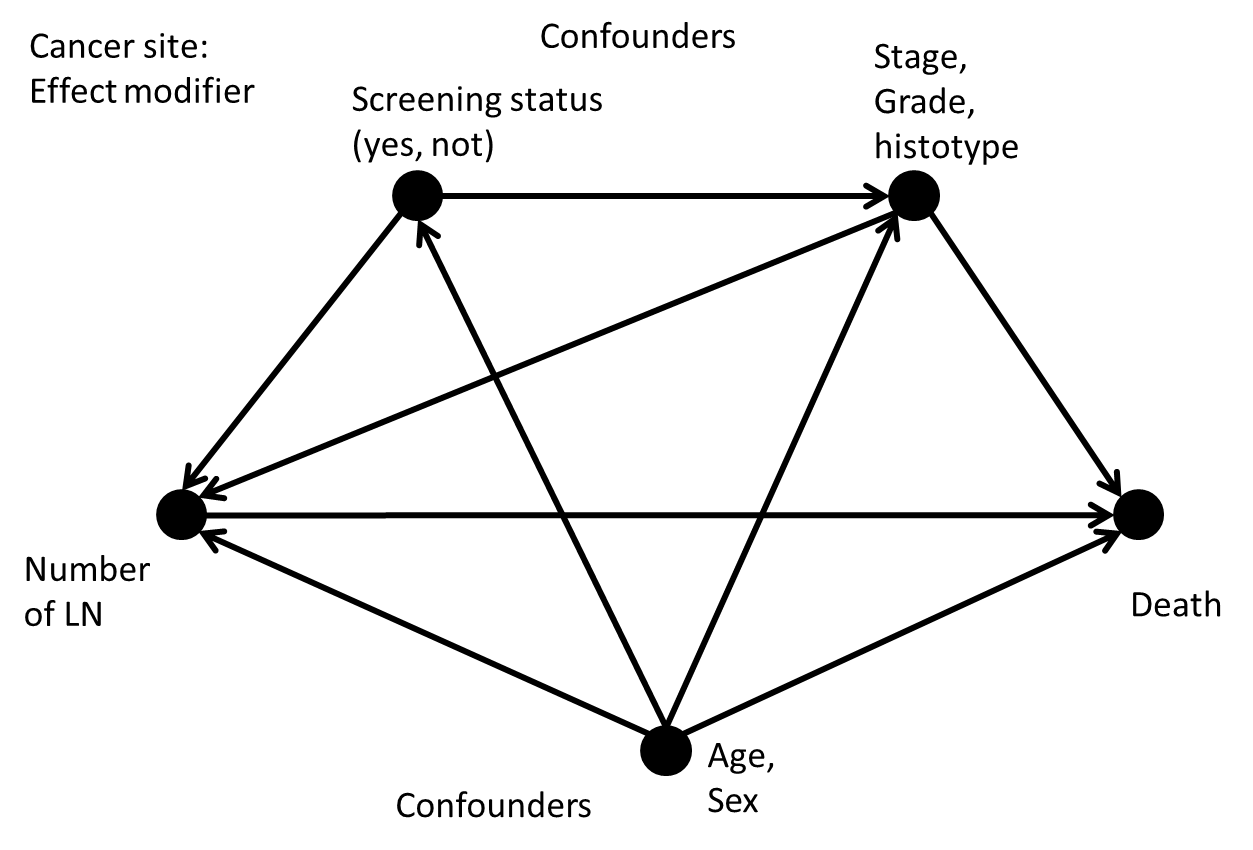


**Legend**

The number of removed lymph nodes is influenced by screening (directly or through a shift to earlier stages), age, sex, and histotype; stage and grade are also determinants of the number of lymph nodes removed and are associated with death; age and sex are possible confounders since they are associated also with death, screening status, stage, and grade; stage, grade, and histotype are possible mediators of the effect of screening on survival; the number of lymph nodes removed may also mediate the effect of stage on mortality. Finally, we observed that cancer site was a strong effect modifier, changing the total effect of the number of removed lymph nodes on survival.

**Multivariable Cox proportional hazard model reporting hazard ratios for cancer site adjusted for age, sex, screening status. Incident colon cancer cases, Emilia-Romagna, Italy, 2000-2012, follow up until December 31, 2017. Check of the proportional hazard assumption for the three colon site groups.**

Supplementary Figure 3. Log-log plot of survival by colon cancer side. Curves are constantly parallel after the first month of follow up, i.e., ln -2.5years of follow up.

Supplementary Figure 4. Comparison of the observed Kaplan–Meier survival curves and Cox proportional hazard predicted survival curves. For all three groups (right, left, and transvers), observed and predicted curves substantially overlap.

**Multivariable Cox proportional hazard model reporting hazard ratios for cancer site adjusted for age, sex, screening status. Incident colon cancer cases, Emilia-Romagna, Italy, 2000-2012, follow up until December 31, 2017. Check of the linearity of the effect of age on survival.**

Supplementary Figure 5. Plots of Martingale residuals computed for age. The plot of Martingale residuals is random, showing no systematic patterns or trends, and the LOESS smoothed curve appears to lie around a horizontal line through zero, supporting the linear component of the age variable correctly describing the effect of age on survival.

**Multivariable Cox proportional hazard models reporting hazard ratios for cancer number of lymph nodes removed by cancer site. Incident colon cancer cases with lymphadenectomy, stages II and III, Emilia-Romagna, Italy, 2000-2012, follow up until December 31, 2017. Check of the proportional hazard assumption for the number of removed lymph nodes.**

Supplementary Figure 6. Log-log plot of survival curves by number of lymph nodes, right-sided cancers. Curves are constantly parallel throughout follow up.

Supplementary Figure 7. Comparison of the observed Kaplan–Meier survival curves and Cox proportional hazard predicted survival curves, right-sided cancers. For all the three groups (<12, 12-21, >21 removed lymph nodes), observed and predicted curves substantially overlap.

Supplementary Figure 8. Log-log plot of survival curves by number of lymph nodes, left-sided cancers. Curves are constantly parallel throughout follow up.

Supplementary Figure 9. Comparison of the observed Kaplan–Meier survival curves and Cox proportional hazard predicted survival curves, left-sided cancers. For all the three groups (<12, 12-21, >21 removed lymph nodes), observed and predicted curves substantially overlap.

Supplementary Table 2. Multivariable Cox proportional hazard model reporting hazard ratios for cancer site adjusted for age, sex, screening status, stage, and grade. Incident colon cancer cases, Emilia-Romagna, Italy, 2000-2012, follow up until December 31, 2017. Number of observations=8,327.

| Factors |  | ***Multivariable analysis*** | | |
| --- | --- | --- | --- | --- |
|  |  | *HR* | *95% CI* | P *value* |
| *(Site) Left* |  | 1 |  |  |
| Right |  | 1.11 | (1.02-1.21) | 0.012 |
| Transverse |  | 1.03 | (0.87-1.22) | 0.696 |
| *Age* |  | 1.05 | (1.04-1.05) | <0.001 |
| *(Sex) Male* |  | 1 |  |  |
| Female |  | 0.70 | (0.65-0.76) | <0.001 |
| *(Screening) Uninvited/ non-attendees* |  | 1 |  |  |
| Screen-detected/interval cancers |  | 0.65 | (0.60-0.71) | <0.001 |
| *(Histological type) Adenocarcinoma* |  | 1 |  |  |
| Carcinoma NOS^a^ |  | 2.09 | (1.25-3.50) | 0.005 |
| Mucinous |  | 1.19 | (1.04-1.36) | 0.013 |
| Other |  | 0.91 | (0.40-2.03) | 0.814 |
| *(Stage) I* |  | 1 |  |  |
| II |  | 1.43 | (1.25-1.64) | <0.001 |
| III |  | 2.51 | (2.20-2.85) | <0.001 |
| IV |  | 11.76 | (10.3-13.37) | <0.001 |
| Unknown |  | 2.32 | (1.74-3.09) | <0.001 |
| *(Grade) G1* |  | 1 |  |  |
| G2 |  | 0.85 | (0.75-0.98) | 0.023 |
| G3-4 |  | 1.31 | (1.13-1.52) | <0.001 |

Supplementary Table 3. Multivariable Cox proportional hazard model reporting hazard ratios for cancer site adjusted for age, sex, screening status, stage, grade, and number of removed lymph nodes. Incident colon cancer cases, Emilia-Romagna, Italy, 2000-2012, follow up until December 31, 2017. Number of observations=8,274.

| Factors |  | ***Multivariable analysis*** | | |
| --- | --- | --- | --- | --- |
|  |  | *HR* | *95% CI* | P *value* |
| *(Site) Left* |  | 1 |  |  |
| Right |  | 1.22 | (1.12-1.33) | <0.001 |
| Transverse |  | 1.06 | (0.90-1.26) | 0.480 |
| *Age* |  | 1.04 | (1.04-1.05) | <0.001 |
| *(Sex) Male* |  | 1 |  |  |
| Female |  | 0.70 | (0.65-0.76) | <0.001 |
| *(Histological type) Adenocarcinoma* |  | 1 |  |  |
| Carcinoma NOS^a^ |  | 1.78 | (1.06-2.98) | 0.029 |
| Mucinous |  | 1.20 | (1.05-1.38) | 0.007 |
| Other |  | 0.75 | (0.34-1.68) | 0.483 |
| *(Screening) Uninvited/ non-attenders* |  | 1 |  |  |
| Screen-detected/interval cancers |  | 0.64 | (0.59-0.69) | <0.001 |
| *(Stage) I* |  | 1 |  |  |
| II |  | 1.75 | (1.51-2.02) | <0.001 |
| III |  | 3.05 | (2.66-3.49) | <0.001 |
| IV |  | 13.61 | (11.93-15.54) | <0.001 |
| Unknown |  | 1.63 | (1.21-2.19) | 0.001 |
| *(Grade) G1* |  | 1 |  |  |
| G2 |  | 0.98 | (0.85-1.12) | 0.731 |
| G3-4 |  | 1.49 | (1.28-1.73) | <0.001 |
| *(LN harvest) <12* |  | 1 |  |  |
| 12-21 |  | 0.82 | (0.74-0.91) | <0.001 |
| >21 |  | 0.69 | (0.61-0.77) | <0.001 |
| Not Removed |  | 1.74 | (1.49-2.04) | <0.001 |

Supplementary Figure 10. Definition of left and right colon as reported in recently published studies.
